# Detection of Hereditary 1,25-Hydroxyvitamin D-Resistant Rickets Caused by Uniparental Disomy of Chromosome 12 Using Genome-Wide Single Nucleotide Polymorphism Array

**DOI:** 10.1371/journal.pone.0131157

**Published:** 2015-07-08

**Authors:** Mayuko Tamura, Tsuyoshi Isojima, Minae Kawashima, Hideki Yoshida, Keiko Yamamoto, Taichi Kitaoka, Noriyuki Namba, Akira Oka, Keiichi Ozono, Katsushi Tokunaga, Sachiko Kitanaka

**Affiliations:** 1 Department of Pediatrics, Graduate School of Medicine, The University of Tokyo, Tokyo, Japan; 2 Department of Human Genetics, Graduate School of Medicine, The University of Tokyo, Tokyo, Japan; 3 Department of Pediatrics, North Medical Center, Kyoto Prefectural University of Medicine, Kyoto, Japan; 4 Department of Pediatrics, Osaka University Graduate School of Medicine, Osaka, Japan; Odense University Hospital, DENMARK

## Abstract

**Context:**

Hereditary 1,25-dihydroxyvitamin D-resistant rickets (HVDRR) is an autosomal recessive disease caused by biallelic mutations in the vitamin D receptor (VDR) gene. No patients have been reported with uniparental disomy (UPD).

**Objective:**

Using genome-wide single nucleotide polymorphism (SNP) array to confirm whether HVDRR was caused by UPD of chromosome 12.

**Materials and Methods:**

A 2-year-old girl with alopecia and short stature and without any family history of consanguinity was diagnosed with HVDRR by typical laboratory data findings and clinical features of rickets. Sequence analysis of *VDR* was performed, and the origin of the homozygous mutation was investigated by target SNP sequencing, short tandem repeat analysis, and genome-wide SNP array.

**Results:**

The patient had a homozygous p.Arg73Ter nonsense mutation. Her mother was heterozygous for the mutation, but her father was negative. We excluded gross deletion of the father’s allele or paternal discordance. Genome-wide SNP array of the family (the patient and her parents) showed complete maternal isodisomy of chromosome 12. She was successfully treated with high-dose oral calcium.

**Conclusions:**

This is the first report of HVDRR caused by UPD, and the third case of complete UPD of chromosome 12, in the published literature. Genome-wide SNP array was useful for detecting isodisomy and the parental origin of the allele. Comprehensive examination of the homozygous state is essential for accurate genetic counseling of recurrence risk and appropriate monitoring for other chromosome 12 related disorders. Furthermore, oral calcium therapy was effective as an initial treatment for rickets in this instance.

## Introduction

Hereditary 1,25-dihydroxyvitamin D (1,25[OH]_2_D)-resistant rickets (HVDRR) (OMIM #277440), also known as vitamin D-dependent rickets type 2A (VDDR 2A), is a rare disorder characterized by early onset rickets, hypocalcemia, and secondary hyperparathyroidism, and alopecia when severe [1]. Patients with HVDRR have high circulating levels of 1,25(OH)_2_D, and are resistant to 1,25(OH)_2_D_3_ and 1α(OH)D_3_ treatment. Elevated 1,25(OH)_2_D levels differentiate HVDRR from 1α-hydroxylase deficiency, which is known as vitamin D-dependent rickets type 1A [2].

HVDRR is caused by mutations in the vitamin D receptor (VDR) gene on chromosome 12q13.11 [3]. HVDRR shows autosomal-recessive inheritance and the patients usually have biallelic mutations in the *VDR* inherited from each parent. Because of the rarity of the disease, most cases arise in consanguineous families and have homozygous mutations [4]. VDR is a member of the steroid/nuclear receptor superfamily of ligand-activated transcription factors, and it is composed of an N-terminal DNA binding domain (DBD) and a C-terminal ligand-binding domain (LBD) [5]. Patients with a mutation in the DBD usually show severe vitamin D resistance associated with alopecia [6], whereas those with a mutation in the LBD show various degrees of vitamin D unresponsiveness and can occasionally respond to high-dose vitamin D. Patients with alopecia showing resistance to high-dose vitamin D therapy usually require intravenous calcium infusions to treat clinically overt rickets at their first presentation [6].

Some recessive disorders have been reported to be caused by uniparental disomy (UPD) of a single parent allele with a mutation [7]. UPD refers to a condition in which both homologues of a chromosomal region or segment are inherited from only one parent. To date, most cases of UPD have been found in imprinting diseases such as Prader-Willi syndrome, and the incidence of UPD of any chromosome is estimated to be as frequent as 1 per 3,500 live births [8,9]. Conversely, UPD causing recessive diseases have only been reported in a limited number of cases. Moreover, no cases have been reported in HVDRR and complete isodisomy of chromosome 12 is extremely rare [7,10,11].

In this report, we used genome-wide single nucleotide polymorphism (SNP) array analysis to determine whether HVDRR was caused by UPD of chromosome 12. Furthermore, we observed the effectiveness of high oral calcium therapy for the treatment of rickets in this severe HVDRR patient.

## Materials and Methods

### Clinical case

**Fig 1:**
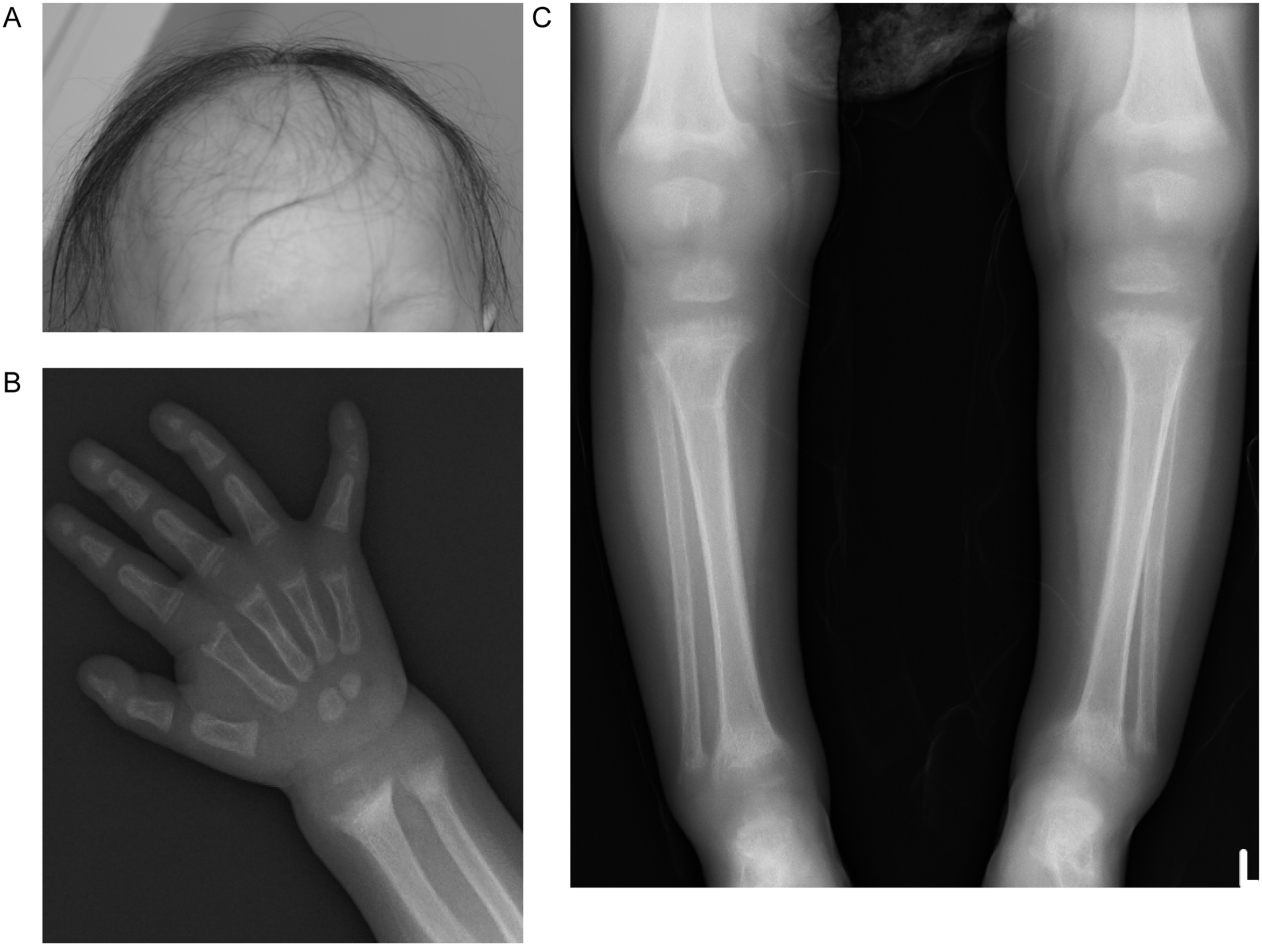
Alopecia and Rickets in the Proband at Presentation. (A) Alopecia. (B,C) A bone roentgenogram of the arm (B) and legs (C) showing cupping, fraying, and flaring at the end of the long bones.

A 2-year 1-month-old girl presented to hospital with fever, at which point she was noted to have short stature, alopecia (Fig 1), and gait instability. Her parents were non-consanguineous and approximately 30 years old when she was born. She had no family history of rickets or unresolved pain. Her mother got a natural conception, and the pregnancy and delivery was uneventful. Her birth weight was 2,868 g (-0.8 standard deviations [SD]), birth length 51 cm (+0.6 SD), gestational age 41 weeks. She had no episode of convulsion and her psychomotor development was normal until she started walking alone at 1 year 3 months of age, but she could not run by age 2. Her body height at presentation was 74.8 cm (−3.5 SD), and her body weight, 9.7 kg (−1.2 SD). She had symptoms of rickets such as bow-legs and enlargement of the limb joints, but had no other external malformation, dysmorphic features, or ataxia. Her verbal developmental quotient (DQ) was 81 and cognitive DQ 94.

Laboratory data revealed hypocalcemia (7.7; reference: 8.5–10.5 mg/dL), hypophosphatemia (3.0; reference: 4.5–6.5 mg/dL), markedly elevated levels of serum alkaline phosphatase (8,891; reference: 300–1,239 IU/L), and intact parathyroid hormone (PTH) levels (576; reference: 10−65 pg/mL). Her serum 1,25(OH)_2_D level was high (137; reference: 20–70 pg/mL) with a normal 25(OH)D level (20.1 ng/mL). Serum levels of fibroblast growth factor 23 (FGF23) were low (<10 pg/mL), and a bone roentgenogram showed characteristic findings of rickets (Fig 1). From these findings, she was clinically diagnosed as having HVDRR.

### VDR gene analysis

We obtained written informed consent for DNA analysis from the parents, and the Ethics Committee of The University of Tokyo approved the study. Genomic DNA was extracted from peripheral white blood cells of the patient and parents using a QIAamp DNA Blood Midi Kit (Qiagen, Hilden, Germany). The entire coding region and exon-intron boundaries of the *VDR* were amplified from the genomic DNA by polymerase chain reaction (PCR) using the specific primers (S1 Table). PCR products were subsequently sequenced using an ABI Prism BigDye Terminator Cycle Sequencing Ready Reaction Kit (PE Applied Biosystems, Foster City, CA) and the forward and reverse primers from the PCR amplification. Direct sequencing in both directions was performed on an autosequencer (PE Applied Biosystems 3130x1, Genetic Analyzer).

### Analysis of common gene polymorphisms

Common SNPs in the *VDR* (rs10735810, rs7975232, rs2853562 rs731236, rs12717991), other genes on chromosome 12 (rs2259820, rs2464196, rs1169289, rs1169288, rs1169301, rs1169304, rs10877012), and other chromosomes (rs4588, rs7041, rs116930, rs1155563, rs2060793, rs3829251, rs6013897, rs6599638, rs10741657, rs12785878, rs17217199) were analyzed by sequencing the PCR products as previously reported [12,13]. Short tandem repeat (STR) analysis was performed using AmpFLSTR Identifiler kit (Identifiler, Applied Biosytems, Foster City, CA, USA), which included 16 STR markers (D8S1179, D21S11, D7S820, CSF1PO, D3S1358, TH01, D13S317, D16S539, D2S1338, D19S433, vWA, TPOX, D18S51, Amelogenin, D5S818, and FGA), according to the manufacturer’s protocol.

### Genome-wide SNP array

Using of the Affymetrix Axiom ASI 1 array (Affymetrix, CA, USA) in accordance with the manufacturer’s instructions, we genotyped a total of 600,307 SNPs for the three individuals. Genotype calls were determined using the Genotyping Console 4.1.4 software with the Birdseed v2 algorithm provided by the manufacture. In addition to data from the patient and her parents, we also used 474 Japanese individuals to ensure reliable genotype calling. Signal intensities for alleles A and B were observed by using Affymetrix Power Tools [14]. B allele frequency was calculated by using of the intensities of both alleles: BAF = B / (A + B).

## Results

### Identification of the *VDR* mutation

**Fig 2:**
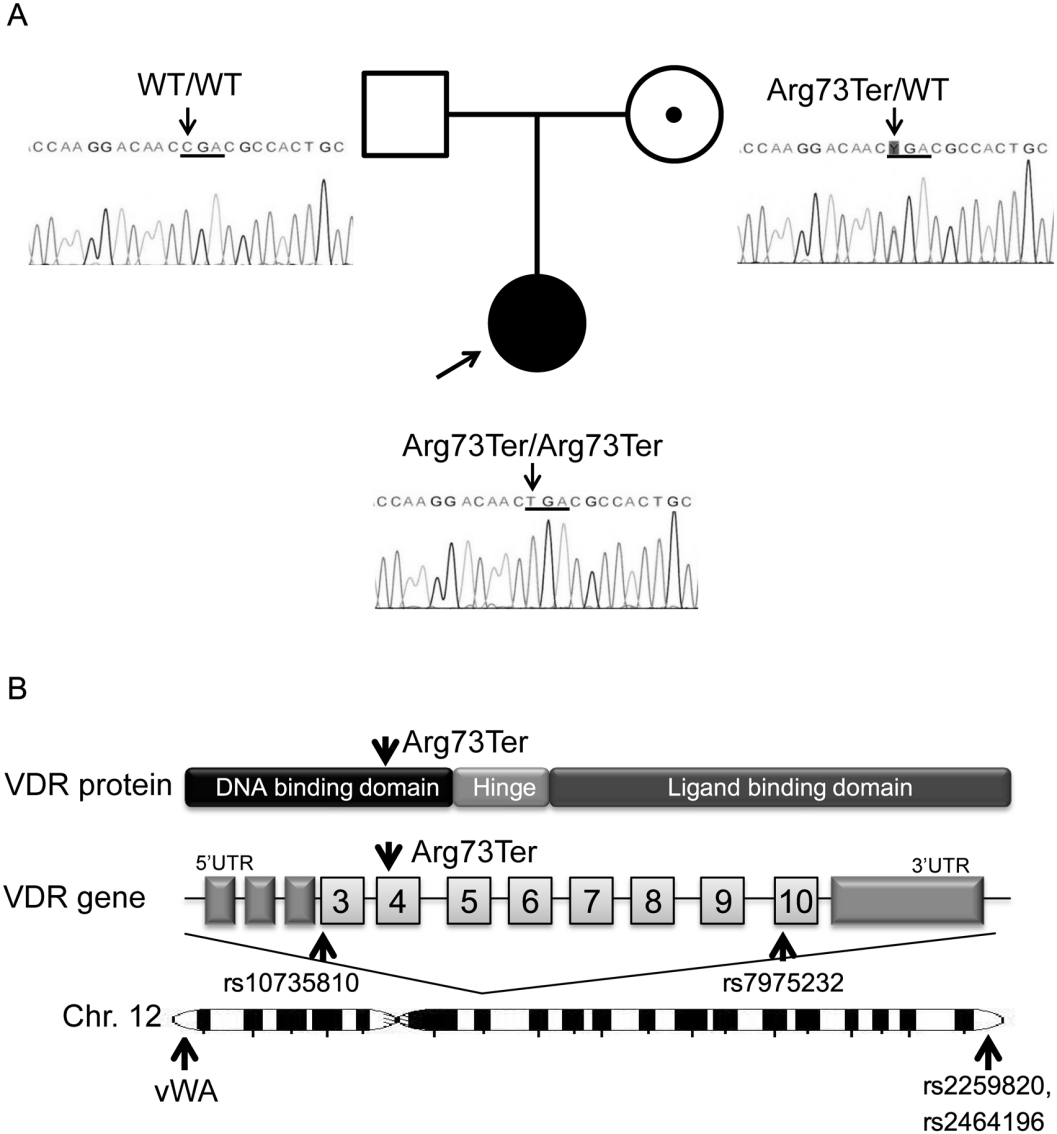
*VDR* Analysis of the Proband and her Parents, with the Position of the Target SNPs on Chromosome 12. (A) Proband pedigree with chromatograms of the *VDR* mutation. The *VDR* analysis showed a homozygous Arg73Ter mutation in the proband, a heterozygous mutation in the mother, and no mutation in the father. Mutations were checked by bidirectional sequencing. A black symbol indicates the proband, a dot symbol indicates a carrier, a square indicates a male, and a circle indicates a female. (B) Diagram of the VDR protein and gene, and chromosome 12 with positions of the mutation and the sequence-analyzed SNPs. The nonsense mutation, Arg73Ter, is located in the DNA binding domain of the VDR protein in exon 4. The indicated common SNPs were analyzed in the family; vWA indicates the position of a marker included in the STR analysis.

Sequencing the *VDR* in the patient revealed a single homozygous base pair substitution, c.217C>A (Fig 2A). This substitution was predicted to result in a nonsense mutation p.Arg73Ter, which is a premature stop codon in the DBD (Fig 2B). This mutation has been reported in 5 other patients with HVDRR and is functionally inactive [15–18]. From these findings, we considered that HVDRR in this patient was caused by a homozygous nonsense mutation in the *VDR*.

### Analysis of the genesis of homozygosity

Most cases of homozygosity among rare mutations are caused by consanguinity, but the parents in this case denied consanguinity; therefore, we performed a genetic analysis of the parents. The mutation was heterozygous in the mother only, and was not present in the father (Fig 2A).

To assess the possibility of a gross deletion around the mutation in the father’s allele, we sequence-analyzed several common target SNPs in the *VDR*, in other genes on chromosome 12, and in other chromosomes studied in our laboratory. Table 1 shows the results of the SNPs which identify the parental origin of the proband’s allele. Two SNPs in the *VDR*, one near the mutation (rs10735810) and another within approximately 20 kb (rs7975232) (Fig 2B), demonstrated homozygous alleles in the proband that were derived only from the mother (Table 1). Moreover, another 2 SNPs located at the opposite end of the long arm of chromosome 12 (rs2259820, rs2464196) (Fig 2B) were also homozygous and derived only from the mother. However, SNPs on chromosomes 4 and 11 showed a normal pattern of allele inheritance from the parents. These results indicated that at least the long arm of chromosome 12 consists of alleles derived only from the mother. G-banding karyotype analysis of the proband revealed a normal 46,XX karyotype without monosomy of 12q. These findings suggested that gross deletion of the father’s allele was unlikely.

**Table 1:** Sequence Analysis of the Common Target SNPs that Could Identify Parental Origin.

Gene	SNP	Location	Proband	Father	Mother
*VDR*	rs10735810	12q13.11	CC	TT	CT
*VDR*	rs7975232	12q13.11	GG	TT	GG
*HNF1A*	rs2259820	12q24.31	CC	TT	CC
*HNF1A*	rs2464196	12q24.31	GG	AA	GG
*NADSYN1*	rs3829251	11q13.4	GA	GG	AA
*GC*	rs7041	4q13.3	TG	TG	GG
*GC*	rs1155563	4q13.3	CT	CC	TT

Next, we assessed the biological paternity by conventional STR analysis. STRs located on chromosomes other than 12 confirmed that the father was the biological father. Interestingly, the STR of a gene located on the short arm of chromosome 12 (vWA; 12p12-pter) showed a homozygous maternal allele (proband 19; father 14, 16; mother 16, 19). Taken together, these findings eliminated the possibility of a gross deletion and paternal discordance, and suggested that *de novo* mutation was unlikely. Finally, maternal UPD of the entire chromosome 12 was suggested.

### Detection of UPD by genome-wide SNP array

**Fig 3:**
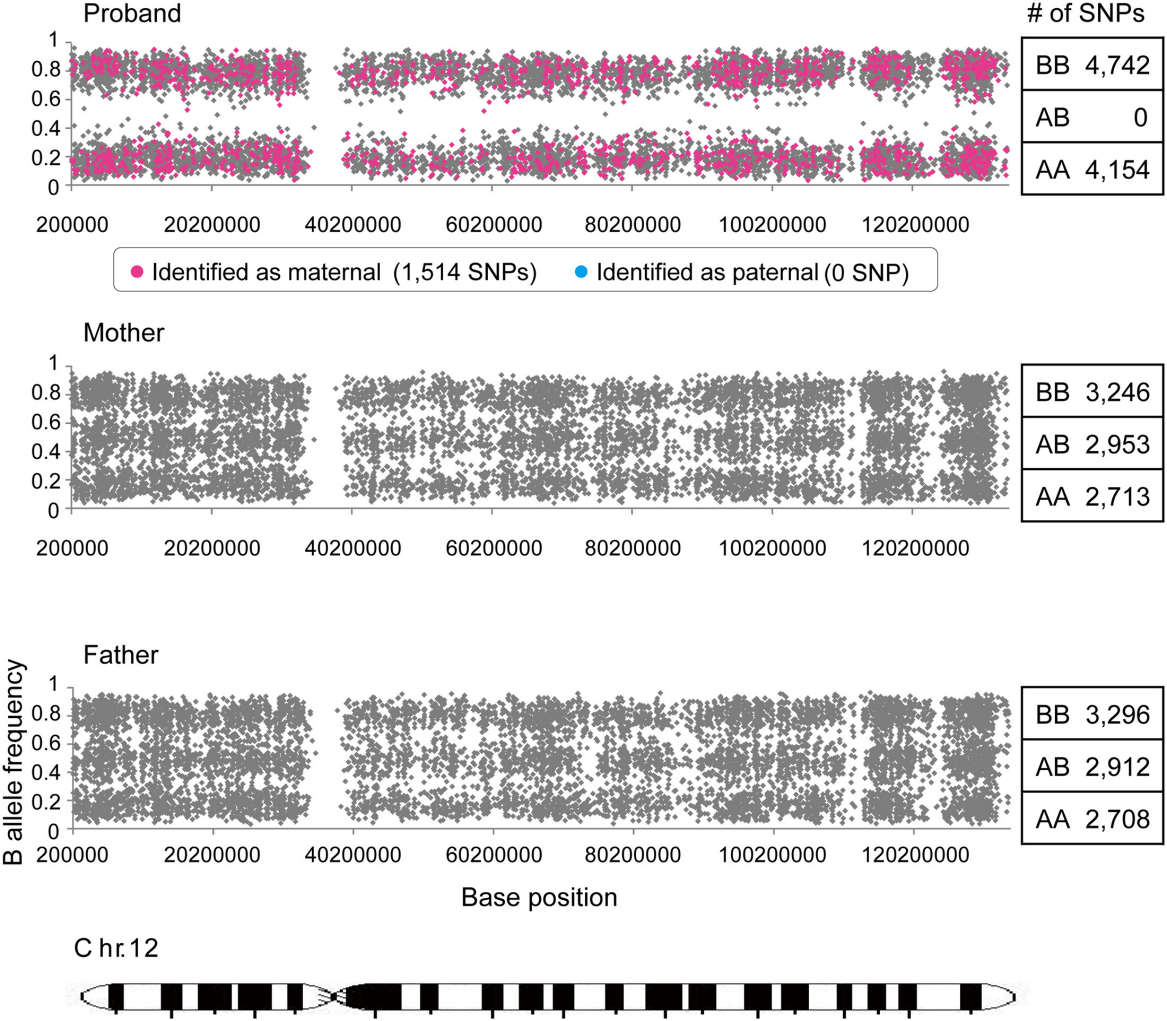
Genome-wide SNP Array Indicating Complete Maternal Isodisomy of Chromosome 12. B allele frequencies of chromosome 12 in the proband, the mother, and the father are shown. Allele segregation in the proband comprised AA (4,154 SNPs) and BB (4,742 SNPs) homozygotes only, and loss of heterozygosity (AB; 0 SNP). The parental allele segregation of chromosome 12 showed a heterozygous pattern. All other chromosomes had normal homo/heterozygous patterns. In the diagram for the proband, the pink spots (1,514 SNPs) represent SNPs identified as maternal, with no evidence of paternal SNPs (which would be blue).

For the evaluation of UPD, we conducted a genome-wide SNP array of the proband and the parents. The overall call rates were 99.47%, 99.64%, and 99.57% for the proband, the father, and the mother, respectively. All chromosomes other than chromosome 12 showed a normal homo/heterozygous pattern. There were 29,197 SNPs on chromosome 12 on the array, of which 13,940 SNPs showed multiple genotypes among the trio of samples (proband, mother, and father). The proband was called homozygous for 13,848 SNPs and heterozygous for 92 SNPs; however, we found that these heterozygous SNPs were miss-calls caused by the genotype calling algorithms, and the proband was considered homozygous for all SNPs on chromosome 12. After linkage disequilibrium pruning (LD pruning) with 474 samples, a total of 8,933 SNPs remained [19,20], except for bad clusters. Fig 3 shows the B allele frequencies for chromosome 12, which represents the distribution of each proband, maternal, and paternal allele. On chromosome 12, the allele segregation revealed to be composed of only homozygous AA and BB combinations, and no AB combinations (loss of heterozygosity). Allele segregation of chromosome 12 showed a heterozygous pattern in her parents. In the proband’s diagram, the pink spots represent the maternal SNPs (1,514 SNPs) and the blue spots, paternal (none) (Fig 3). It was obvious that all of the homozygous SNPs on chromosome 12 derived from the mother. Moreover, the 2 alleles in the proband were 100% identical to those in the mother by identical-by-descent analysis, whereas none were identical to those in the father [21]. The signal intensity of chromosome 12 was sufficient to conclude that the chromosome was diploid, compared with other chromosomes. Thus, we concluded that the proband had complete maternal uniparental isodisomy of chromosome 12 with a nonsense mutation.

### Clinical course

**Fig 4:**
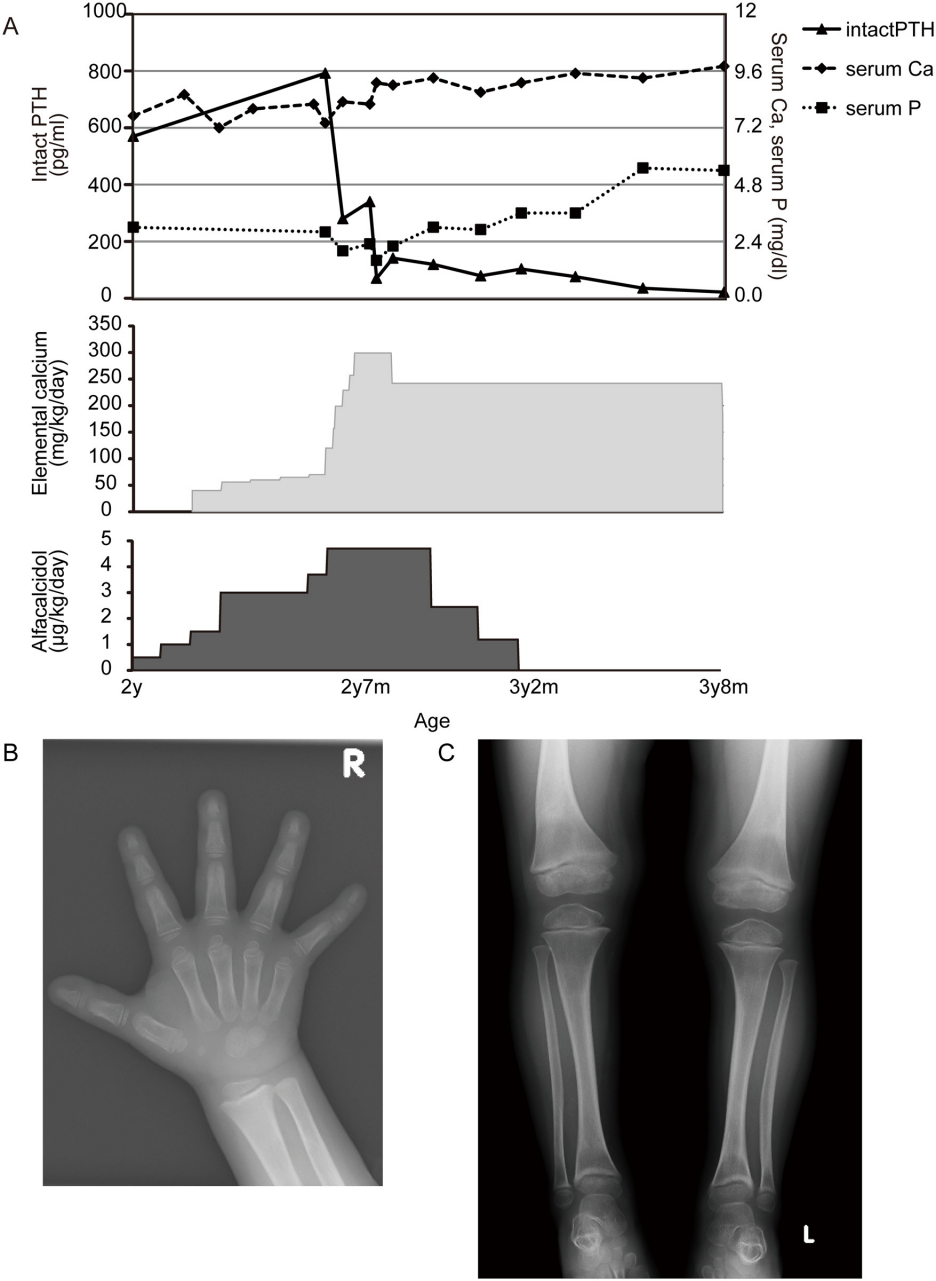
After Treatment with High-dose Oral Calcium Supplementation. (A) Treatment course and laboratory data are shown. (B) Bone roentgenogram at 3 years and 8 months of age, showing markedly improved signs of rickets.

Initial treatment with increasing amounts of alfacalcidol up to 5 μgkg/day with oral calcium 60 mg/kg/day failed to improve her symptoms. After detecting the *VDR* mutation, she was admitted to another hospital for further treatment. After increasing her oral elemental calcium (calcium lactate) dose to 300 mg/kg/day (divided 3 times), her serum calcium and PTH levels improved (Fig 4A). The oral calcium was subsequently reduced to 240 mg/kg/day when high urinary calcium excretion started; the alfacalcidol dose was stopped because it was considered ineffective based on the genetic analysis. After 12 months of therapy, her laboratory data, including alkaline phosphatase levels, had normalized, her height gain improved, she started to run, and a repeat bone roentgenogram showed an improvement in the features of rickets (Fig 4B and 4C). Her most recent 1,25(OH)_2_D level was 20 pg/mL, whereas her FGF23 was 12 pg/mL, and her urine calcium/creatinine ratio was 0.5. Although her rickets improved, alopecia has remained. Her amblyopia was noticed at age three.

## Discussion

This is the first report of an HVDRR caused by UPD of the mutant allele. We also found that genome-wide SNP array was useful for the detection of the complete isodisomy. We first noted an unusual homozygous state in this non-consanguineous family, and found that only the mother was heterozygous for the mutation. Such non-Mendelian inheritance implied the possibility of gross deletion of the father’s allele, non-paternity, UPD, or some *de novo* mutation. However, common SNP sequencing and STR analysis suggested a homozygous chromosome 12 and confirmed paternity, whereas subsequent G-band karyotyping excluded gross deletion of chromosome 12. Finally, the genome-wide SNP array confirmed that complete maternal isodisomy of chromosome 12 was present. This molecular diagnosis is fundamental to ensure accurate genetic counseling of the risk of recurrence in the next child, which would be far less than 25%, as would typically be the case for a recessive disorder.

Uniparental isodisomy refers the inheritance of two identical copies of the same chromosome, and different from uniparental heterodisomy, which is the inheritance of the two different homologous chromosomes from one parent. Both of isodisomy and heterodisomy can cause genome imprinting disorders, but only uniparental isodisomy can cause recessive disorders if the chromosome harbors a disease-causing mutation. As a result, the mutated allele of her mother became homozygous in the proband to present non-Mendelian inheritance of the autosomal recessive disease, HVDRR. Although high maternal age is suggested as a risk factor for maternal UPD in imprinting disorders, it is mostly meiotic nondisjunction causing heterodisomy and differs for this case of isodisomy [22].

For the molecular detection of UPD, microsatellite marker analysis has been used conventionally, which needs parents’ samples to demonstrate UPD [23]. Recently, the new technology of genome-wide SNP array had facilitated the detection of UPD [9,24–26]. SNP array can detect isodisomy by loss of heterozygosity of the segment or whole chromosome without parents’ samples. It is sufficient to demonstrate UPD if there are no chromosomal aberration by cytogenetic analysis. More recently, using a signal intensity analysis of the SNP array, it is possible to evaluate the copy number changes, so that even chromosome banding is unnecessary. Furthermore, we have shown it possible to detect the origin of the homozygous chromosome by analyzing together with the parents’ allele. For example, if the proband’s allele is AA, mother’s AA or AB, and father’s allele BB, the proband’s allele reveals to be derived only from the mother. In this point, our study is distinguished from others.

There are only two reported cases of chromosome 12 UPD leading to recessive disorders, one maternal and one paternal [27,28], making this the third case. The occurrence of UPD in each chromosome does not appear to be equivalent, and is rare in chromosome 12. UPD of chromosomes 6, 7, 11, 14, 15, and 20 has been reported to cause imprinting disorders; however, none is known for chromosome 12. Although we show the limited period of one case, the fact that our patient shows no other than typical symptoms of HVDRR by the age of 3, suggests that there are no genomic imprinting diseases caused by UPD of maternal chromosome 12. Moreover, it is also suggested that the UPD allele in the proband contained no other disease-causing mutations that would present by the age of 3. In theory, there may be at least 3 other recessive disease-causing mutations in this homozygous chromosome (average mutations > 200 per genome, maternal chromosome 12 constitution approximately 2%) [26,29]. The discovery of UPD will require careful observation for late manifestations of other chromosome 12 related genetic disorders. Furthermore, we consider from this study that, although there are relatively few reports of recessive diseases caused by UPD, this phenomenon may be more frequent and only have not analyzed.

Our patient with deleterious *VDR* mutation was successfully treated with high-dose oral calcium. She had no trouble taking large doses of calcium lactate orally after every meal. Most of the reported cases with severe HVDRR have required intravenous calcium infusions for initial treatment, which often lead to prolonged hospitalizations and increased risks of catheter-related complications [6,30]. Although oral calcium with vitamin D therapy is reportedly effective in some cases, it is mainly reserved for use as a maintenance therapy [18,31]. Although this is one unusual case with UPD, the clinical course of our case suggested that oral calcium therapy is effective in the initial treatment in some cases of severe HVDRR.

Among the intestinal calcium absorption, active transport of calcium through calcium transporters is induced by VDR and 1,25(OH)_2_D. On the other hand, high dietary calcium with lactose can induce passive transport, which is considered to be VDR-independent [32,33]. It has been shown in the VDR-null mice that bone abnormalities can be rescued by high calcium diet [34,35]. We consider that although the mutant VDR in this case is inactive, high calcium diet induced passive calcium transport at the intestine and improved her rickets. However, alopecia is considered as VDR-mediated but not calcium-mediated phenotype, and was unresponsive to high calcium diet, which was also similar to the observations on VDR-null mice [36].

## Conclusions

HVDRR in this case was caused by a rare and complete UPD of maternal chromosome 12 with a *VDR* mutation. Genome-wide SNP array helped to detect the isodisomy and parental origin of the allele. Such comprehensive examination of the homozygous state is essential for accurate genetic counseling of recurrence risk and appropriate monitoring for other chromosome 12 related disorders. The treatment course suggested that oral calcium therapy is effective as an initial treatment for rickets in some cases with severe HVDRR.

## Supporting Information

S1 Table Primers and PCR conditions used to amplify the coding region of *VDR*. (DOCX)
