# Supplementary material for: Detection of Hereditary 1,25-Hydroxyvitamin D-Resistant Rickets Caused by Uniparental Disomy of Chromosome 12 Using Genome-Wide Single Nucleotide Polymorphism Array
Source: PLoS One. 2015 Jul 8;10(7):e0131157. doi: 10.1371/journal.pone.0131157 (PMC4496068; doi:10.1371/journal.pone.0131157)
Supplement: S1 Table — (DOCX) [file pone.0131157.s001.docx]

S1 Table. Primers and PCR conditions used to amplify the coding region of *VDR*.

| Primer | Sequence 5’-3’ | Product size (bp) | Annealing temperature |
| --- | --- | --- | --- |
| VDRe3F | AGCTGGCCCTGGCACTGACTCTGGCTCT | 267 | 60 |
| VDRe3R | ATGGAAACACCTTGCTTCTTCTCCCTC |  |  |
| VDRe4F | TTGGAGAAATGGAGACCAGG | 327 | 60 |
| VDRe4R | AGACCCTCTGCCCAAACTT |  |  |
| VDRe5-6F | TAAAGCCCCTCCTATCTTGG | 648 | 60 |
| VDRe5-6R | TTCCATTAGGGAGCCTTCCA |  |  |
| VDRe7F | ACCAGGTTTTTGCAGGGTCT | 338 | 60 |
| VDRe7R | GGTGGATGAGTGATCTCCAA |  |  |
| VDRe8-9F | AAAGACCCAGGGTGCATGTT | 699 | 60 |
| VDRe8-9R | TTTGCTACGTCTCCCTTCAG |  |  |
| VDRe10F | TTGAGTGTCTGTGTGGGTGG | 866 | 60 |
| VDRe10R | GGGCAATGGGATGTTGGTGG |  |  |
